# Chronic Cerebellar Meningoencephalitis in Adulthood: A Report of a Rare Case

**DOI:** 10.7759/cureus.59782

**Published:** 2024-05-07

**Authors:** Vladislav Velchev, Stefan Burev, Dimo Yankov, Stela Petrova, Petar-Preslav Petrov, Remzi R Hyusein, Plamen Penchev

**Affiliations:** 1 Faculty of Medicine, Medical University of Plovdiv, Plovdiv, BGR; 2 Department of Neurological Surgery, University Hospital "St. Ivan Rilski", Sofia, BGR; 3 Department of General and Clinical Pathology, University Multi-profile Hospital for Active Treatment and Emergency Medicine (UMHATEM) - Pirogov, Sofia, BGR; 4 Department of Anatomy, Histology, and Embryology, Medical University of Plovdiv, Plovdiv, BGR; 5 Faculty of Medicine, Medical University - Sofia, Sofia, BGR

**Keywords:** neurosurgery, meningoencephalitis, suboccipital craniotomy, cerebellitis, surgical case report

## Abstract

The development of meningoencephalitis is a result of an inflammation of the meninges and the brain, which can cause neurological sequelae. Cerebellar meningoencephalitis in adult patients is extremely rare and requires special diagnostic approaches. The aim of this report is to present a rare case of meningoencephalitis and evaluate the diagnostic and therapeutic techniques. We present a 45-year-old male patient who has entered the neurosurgery clinic with a severe headache lasting for a month. Neurological status determines intracranial hypertension. Magnetic resonance tomography (MRT) showed evidence of hyperintense lesions with homogenous enhancement in the right hemisphere of the cerebellum. The patient underwent a suboccipital paramedian craniotomy to excise the lesions and for the pathohistological examination of the biopsy material. Biopsy examination found sections expressing an infection process causing chronic meningoencephalitis in the right hemisphere of the cerebellum. The patient was treated postoperatively with cephalothin 2 g every 12 hours for 14 days. Follow-up examinations proved a relief of the symptoms. Meningoencephalitis of the cerebellum and the meninges is a complication that may occur in adulthood, and surgical excision, biopsy examination, and antibiotic therapy are promising methods for managing the disease.

## Introduction

The complex inflammation of the brain parenchyma and the meninges, termed meningoencephalitis, is a rare disease triggered by diverse factors and creates a high risk of lasting neurological sequelae. Nearly half of the cases worldwide remain undetermined of origin [1].

Meningoencephalitis occurring in the cerebellum is a rare condition usually diagnosed in children having severe infectious diseases. Few cases of adults with cerebellitis have been documented [2].

The potential factors for the development of the disease are pathogenic microorganisms such as viruses, bacteria, and fungi, parasites, and autoimmunity. By 2023, the mortality rates of elderly patients vary from 11% to 25%, whereas 15%-25% of survivors face functional disabilities [3]. Clinical manifestations of meningitis include fever, neck stiffness, and an altered mental status [4].

The aim of the article is to present a rare case of meningoencephalitis in the right posterior fossa of a 45-year-old male patient and evaluate the diagnostic and therapeutic techniques.

## Case presentation

**Figure 1:**
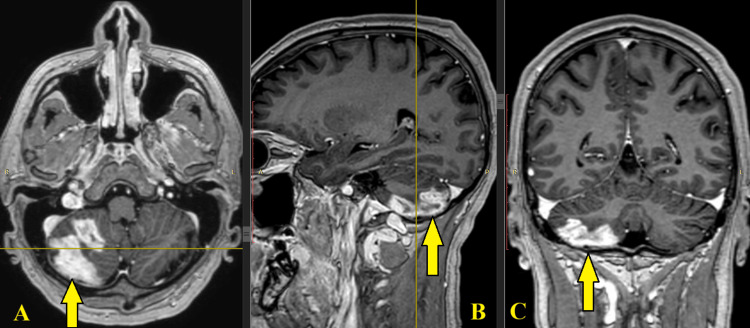
Preoperative MRT of meningoencephalitis in the right posterior cranial fossa (arrows) A: axial plane, B: sagittal plane, C: coronal plane Notice the abnormal lesions in the right hemisphere of the cerebellum MRT: magnetic resonance tomography

We present a 45-year-old male patient who experienced severe long-lasting headaches for one month. After consultations with a neurologist and a neurosurgeon, the patient has entered the neurosurgery clinic for further examinations. Neurological status determined intracranial hypertension and mild coordination deficits. Magnetic resonance tomography (MRT) showed evidence of hyperintense lesions with homogenous enhancement in the right hemisphere of the cerebellum (Figure 1).

The patient underwent surgery to excise the lesions and for the pathohistological examination of the biopsy material. The patient was turned to the left side in a park bench position. Under general anesthesia, a suboccipital paramedian craniotomy from the right side was performed. After durotomy, a lesion has been discovered covering the folia of the right cerebellar hemisphere and the pia and subarachnoid matters. The surgery was successful, and postoperative complications were not observed. The patient was mobilized on the day after the intervention. He was treated postoperatively with a broad-spectrum antibiotic (cephalothin 2 g) every 12 hours.

**Figure 2:**
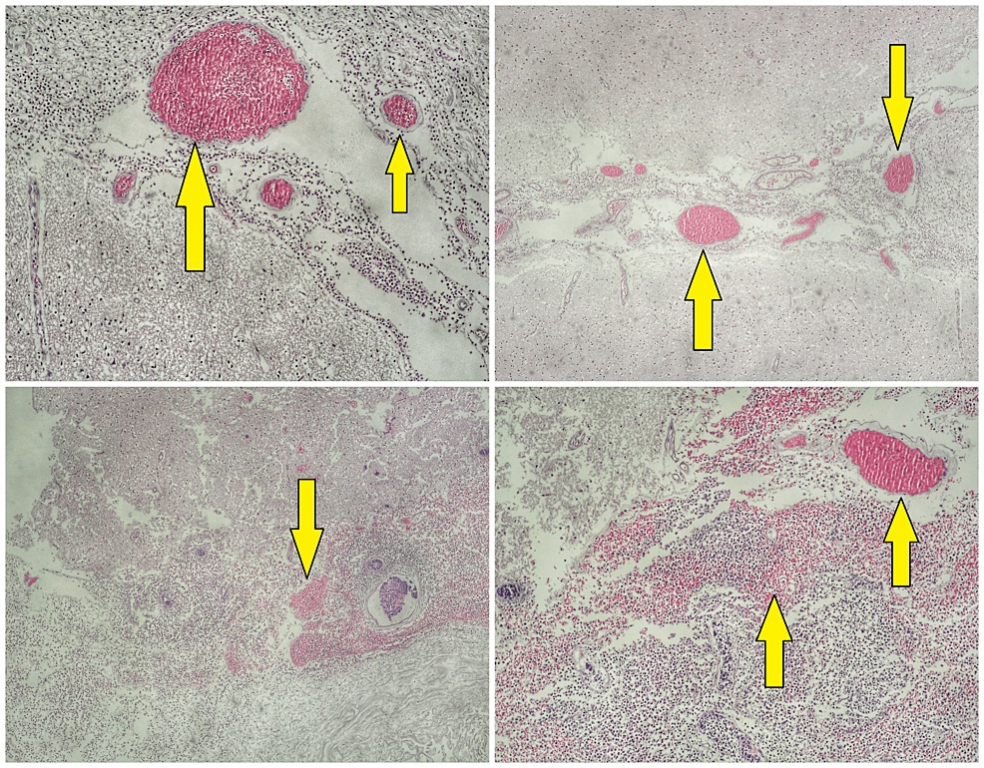
Cerebral cortex in spaces with abundant fibro-purulent inflammatory infiltrate rich in neutrophilic leukocytes (arrows)

Biopsy examination of the lesion identified 5-7 materials with a diameter of 1-3 mm containing parts of the cerebral cortex expressing signs of chronic meningoencephalitis (Figure 2). The matter was characterized by abundant fibro-purulent inflammatory infiltrate rich in neutrophilic leukocytes involving both the meninges and cerebral cortex and white matter forming abscesses, brain edema, and an excess of blood in dilated vessels.

Cytological examination of the cerebrospinal fluid (CSF) revealed the following results (Table 1). No atypical cells were isolated. The microorganism causing the infectious process could not be identified.

**Table 1:** Cytological examination of the cerebrospinal fluid

Parameter	Value
Neutrophils	0.01
Eosinophils	0
Reticulum cells	0
Mononuclear cells	0.04
Macrophages	0
Lymphocytes	0.95
Atypical cells	0
Activated lymphocytes	0
Plexus cells	0
Plasma cells	0
Blast cells	0
Tumor cells	0
Ependymal cells	0

The patient was diagnosed with chronic meningoencephalitis. The pathogen was sporadic. Postoperatively, he received a 14-day antibiotic course with cephalothin 2 g every 12 hours. The patient's symptoms regarding the severe headache were relieved, and fine coordination was restored. Follow-up examinations proved a significant improvement.

## Discussion

Diagnostic approaches

Cerebellar meningoencephalitis in adult patients is extremely rare and requires an individual approach to diagnosing and treatment. Diagnostic methods include microbiological tests of the cerebrospinal fluid and encephalography [5]. Although magnetic resonance imaging is the established standard for detecting central nervous system lesions, it is not necessarily diagnostic of tumor formation [6]. As the brain expresses a few unique characteristics such as the absence of lymphatics and capillaries with the presence of cerebrospinal fluid, the brain reacts to various infections and neoplasms in a limited and stereotypical way. Magnetic resonance tomography (MRT) presents a nonspecific picture of the abnormalities but is precise in localizing and differentiating inflammatory diseases and tumor formations [7]. On MRT, meningoencephalitis presents as hyperintense lesions with homogenous enhancement. Metastatic neoplasms could lead to meningitis characterized by dural enhancement [7]. Due to the similarities of carcinomas and meningoencephalitis, the patient was properly diagnosed only after the biopsy examination.

As the pathogenic factor in half of the patients is undefined, it is essential that microbiological tests of the cerebrospinal fluid (CSF) and pathohistological biopsy examinations are done [1]. The most frequent microorganisms inducing meningoencephalitis in Europe have been documented to be the tick-borne encephalitis virus (TBEV) and *Streptococcus pneumoniae* [8]. In both infectious and neoplastic disorders, the content of CSF exhibits an increased amount of proteins, but the lack of tumor cells in CSF and biopsy contradicts the carcinoma diagnosis [6]. The biopsy examination in this case presented a chronic infection process characterized by abundant fibro-purulent inflammatory infiltrate rich in neutrophilic leukocytes causing chronic meningoencephalitis.

Clinical manifestations

The differential diagnosis of encephalitis and meningitis includes bacterial, viral, and autoimmune factors [9]. The classic features of the disease have been reviewed and summarized in Table 2 and compared to the more complex meningoencephalitis [10].

**Table 2:** Comparative characteristics of meningitis, encephalitis, and meningoencephalitis HSV: herpes simplex virus, VZV: varicella zoster virus, tick-borne encephalitis virus

	Meningitis	Meningoencephalitis	Encephalitis
Affected space	Subarachnoid space	Subarachnoid space and brain parenchyma	Brain parenchyma
Fever, leukocytosis	Yes	Yes	Yes
Nuchal rigidity	Yes	Yes	No
Photophobia	Yes	Yes	No
Mental alteration	Sometimes	Often	Usually
Seizures	Rare	Rare	Often
Typical pathogens	*Streptococcus pneumoniae*, *Neisseria meningitidis*, *Haemophilus influenzae*	*Streptococcus pneumoniae*, HSV, VZV, TBEV, *Listeria* spp.	HSV, VZV, TBEV, enteroviruses

The clinical picture of the disease presents with nausea, headaches, and ataxia in 80% of patients, whereas 29% experience alterations in their consciousness [11]. Coordination and fine motoric movements may suffer as the corticocerebellar pathway and the dental nucleus could be damaged [2].

In our case, the pathogen is sporadic. However, the patient in this case matches the clinical manifestations of chronic meningoencephalitis regarding the severe headaches and the mild coordination deficits. The pathology did not cause any mental alterations and pronounced photophobia because treatment was initiated on time.

Treatment

Despite the fact that antibiotic and antiviral medications are required for the treatment of meningitis and encephalitis, surgical excision of the lesions and decompression are suitable in cases of chronic inflammation, cranial hypertension, and the potential development of a herniation [12]. Yan et al. [13] have documented two cases of surgically operated encephalitis aiming to prevent an uncal herniation. Decompression craniotomy is proven to have promising relieving effects on patients of this disease with refractory intracranial hypertension. Choucha et al. [14] have successfully treated adult cases of meningoencephalitis through this method. Surgical excision was the selected method in this case due to the hypertension in the right posterior fossa causing the severe headaches.

A suboccipital paramedian craniotomy is a prolonged intervention of high precision and complexity, due to the underlying critical anatomical structures [15]. It is compatible with the treatment of cerebellar encephalitis. Amador et al. [16] have reported a case of meningoencephalitis in the right posterior fossa characterized by a swollen cerebellar hemisphere, degradation of the white and grey matter, and cloudy leptomeninges, which was managed with suboccipital paramedian craniotomy.

Relying on the stated advantages of the surgical intervention, the most secure and effective management of the case of cerebellar meningoencephalitis in this adulthood case is the operative excision, biopsy of the lesion, and antibiotic treatment.

## Conclusions

Meningoencephalitis engaging the cerebellum in adulthood is a rare disease that presents with headaches, nausea, and mild coordination neurodeficits. We recommend proper imaging studies as well as microbiological examinations, which are essential methods in acquiring the differential diagnosis of the disease. Surgical treatment with suboccipital paramedian craniotomy in combination with postoperative antibiotic treatment can be an effective method for treating these pathologies.
